# Genetic architecture of innate and adaptive immune cells in pigs

**DOI:** 10.3389/fimmu.2023.1058346

**Published:** 2023-02-06

**Authors:** Maria Ballester, Teodor Jové-Juncà, Afra Pascual, Sergi López-Serrano, Daniel Crespo-Piazuelo, Carles Hernández-Banqué, Olga González-Rodríguez, Yuliaxis Ramayo-Caldas, Raquel Quintanilla

**Affiliations:** ^1^ Animal Breeding and Genetics Program, Institute of Agrifood Research and Technology (IRTA), Torre Marimon, Caldes de Montbui, Spain; ^2^ Unitat mixta d’Investigació IRTA-UAB en Sanitat Animal, Centre de Recerca en Sanitat Animal (CReSA), Universitat Autònoma de Barcelona (UAB), Bellaterra, Catalonia, Spain; ^3^ Institute of Agrifood Research and Technology (IRTA), Programa de Sanitat Animal, Centre de Recerca en Sanitat Animal (CReSA), Universitat Autònoma de Barcelona (UAB), Bellaterra, Catalonia, Spain

**Keywords:** immunocompentence, immune cells, RNA-Seq, biomodel, γδ T cells, pig

## Abstract

Pig industry is facing new challenges that make necessary to reorient breeding programs to produce more robust and resilient pig populations. The aim of the present work was to study the genetic determinism of lymphocyte subpopulations in the peripheral blood of pigs and identify genomic regions and biomarkers associated to them. For this purpose, we stained peripheral blood mononuclear cells to measure ten immune-cell-related traits including the relative abundance of different populations of lymphocytes, the proportions of CD4^+^ T cells and CD8^+^ T cells, and the ratio of CD4^+^/CD8^+^ T cells from 391 healthy Duroc piglets aged 8 weeks. Medium to high heritabilities were observed for the ten immune-cell-related traits and significant genetic correlations were obtained between the proportion of some lymphocytes populations. A genome-wide association study pointed out 32 SNPs located at four chromosomal regions on pig chromosomes SSC3, SSC5, SSC8, and SSCX as significantly associated to T-helper cells, memory T-helper cells and γδ T cells. Several genes previously identified in human association studies for the same or related traits were located in the associated regions, and were proposed as candidate genes to explain the variation of T cell populations such as *CD4, CD8A, CD8B, KLRC2, RMND5A* and *VPS24*. The transcriptome analysis of whole blood samples from animals with extreme proportions of γδ T, T-helper and memory T-helper cells identified differentially expressed genes (*CAPG, TCF7L1, KLRD1* and *CD4*) located into the associated regions. In addition, differentially expressed genes specific of different T cells subpopulations were identified such as *SOX13* and *WC1* genes for γδ T cells. Our results enhance the knowledge about the genetic control of lymphocyte traits that could be considered to optimize the induction of immune responses to vaccines against pathogens. Furthermore, they open the possibility of applying effective selection programs for improving immunocompetence in pigs and support the use of the pig as a very reliable human biomedical model.

## 1 Introduction

Pork is one of the most consumed meats worldwide. The industrialization and specialization of the swine production coupled with the ever-increasing movement of pigs and pork products at national and international scales facilitate the appearance and spread of endemic and emerging pathogens which causes significant economic losses to the swine industry. Furthermore, the restriction in the use of antimicrobials, due to the appearance of drug-resistant bacteria, and the threats of climate change, stresses the need for guiding breeding programs to produce more robust and disease resistant pig populations (1). Along with conventional methods such as vaccination and strict biosecurity measures, improving the overall immunocompetence of healthy animals is a good approach to prevent and control infectious pathogens (2, 3).

In this context, immunity traits (ITs) are considered biologically relevant parameters to measure immunocompetence (4). ITs may be divided in the two major components of the immune system, the innate or natural immunity and the acquired or adaptive immunity. The innate immunity is composed by non-specific components that constitute the first line of host defence and recognize a wide range of pathogens through a restricted set of pattern recognition receptors. Cell types involved in innate immunity include Natural Killer (NK) cells, dendritic cells, monocytes or γδ T cells (5). In contrast, specialized cells of the acquired immune system can provide immunological memory after an initial response mediated by a very large number of specific receptors for antigens present in pathogens (6). While T lymphocytes are the effectors of adaptive cellular immune responses, antibody-producing cells, B lymphocytes, mediate adaptive humoral immunity (6).

The immune system has a high degree of interindividual variation that is controlled by genetic and environmental factors (7). However, leukocyte subsets, mostly the adaptive ones, are highly affected by genetics (8–10). Genetic studies have estimated moderate to high heritabilities for both innate (e.g. NK cells and γδ T cells) and adaptive (e.g. CD4^+^ T lymphocytes, CD8^+^ T lymphocytes and B lymphocytes) immune cells under different conditions in pigs (10–12), suggesting that selection for those traits is a plausible strategy for the swine industry (13). Furthermore, the development of high-density genotyping arrays and in-depth immune phenotyping techniques have allowed the identification of candidate genes and genetic variants associated with the phenotypic variation of immune cells in humans (9, 14) and pigs (15, 16). However, genome-wide association studies (GWAS) for innate and adaptive immune cells in pigs are still scarce. The few studies on this topic have been focused on identifying genetic variants that affect T-cell subpopulations in animals vaccinated at 21 days of age with live classical swine fever (CSF) vaccine (15, 16).

The objective of this work was to study the genetic determinism of both innate and adaptive immune cells traits in healthy pigs of a Duroc commercial line by estimating their genetic parameters and identifying genomic regions, candidate genes and genetic markers associated to their phenotypic variation. Finally, knowing the relevance of the pig as a human biomodel, a comparison between both species was performed with the significantly associated regions found in pigs.

## 2 Materials and methods

### 2.1 Ethics approval

All experimental procedures with pigs were performed according to the Spanish Policy for Animal Protection RD 53/2013, which meets the European Union Directive 2010/63/EU about the protection of animals used in experimentation. The experimental protocol was approved by the Ethical Committee of the Institut de Recerca i Tecnologia Agroalimentàries (IRTA).

### 2.2 Animal material

A total of 391 healthy piglets aged 60 ± 8 days (males and females) belonging to a commercial Duroc line were used in this study. All animals were vaccinated at weaning (aged ~26 days old) against PCV2 with Porcilis^®^ PCV. The pigs came from six batches (72 ± 1 animals per batch) and were raised in the same farm and *ad libitum* fed with a commercial cereal-based diet. All animals were apparently healthy, without any observable signs of infection.

Blood was collected *via* the external jugular vein into vacutainer tubes with anticoagulants (Sangüesa S.A., Spain), which required the restraint of the animals but not their sedation. Genomic DNA was extracted from blood samples using the NucleoSpin^®^ Blood (Macherey-Nagel, Germany). DNA concentration and purity were measured in a Nanodrop ND-1000 spectrophotometer.

### 2.3 Immunophenotyping

Peripheral blood mononuclear cells (PBMCs) isolated from heparinized peripheral blood by density-gradient centrifugation with Histopaque-1077 (Sigma, Spain) and previously stored at -80°C from a study for γδ T-lymphocyte quantification (12), were herein employed for immune phenotyping. Frozen PBMCs were thawed and resuspended in RPMI 1640 medium supplemented with 5% Foetal Bovine Serum (FBS) (Sigma, Spain), 1% Penicillin-Streptomycin (10,000 U/ml - 10 mg/mL) and 1% L-Glutamine (200 mM) (Cultek, Spain) after centrifugation at 450 g at 4°C for 10 min.

Cell concentration was adjusted using RPMI to obtain around 800,000 cells per each sample. Once adjusted, all samples in duplicate were viability stained (Live-or-dyeTM Aqua 405, Biotium, Fremont, CA, USA) diluted in PBS for 25 min at 4°C following the manufacturer’s indications. After two washes, PBMCs were labelled with the primary-conjugated antibody mixture in PBS-1%FBS for 25 min at 4°C (**Supplementary Table 1**).

After two washes with 1xPBS-1% FBS at 4°C, cells were resuspended in 1xPBS-1% FBS and analysed by flow cytometry using the MACSQuant Analyzer 10 Flow cytometer (Miltenyi Biotec GmbH, Bergisch Gladbach, Germany) and the Flowlogic™ software v7.3 (Inivai Technologies, Melbourne, Australia). Beside test samples, unstained cells, stained samples with sole viability marker, isotypes for each antibody subclass, and Fluorescence Minus One (FMO) stained samples were included as controls to adjust the analysis and discard false positive results.

The gating strategy per each animal used for this calculation is shown in **Figure S1**. Absolute number of events were used to calculate percentages of lymphocyte populations depending on PBMCs gate. After discarding the dead cells gated by the use of the viability staining, the following cell subsets were quantified: B lymphocytes (CD21^+^), T lymphocytes (CD3^+^), natural killer (NK) cells (CD3^-^CD21^-^CD8^+^), cytotoxic (CTL) T cells (CD3^+^CD4^-^CD8^+^), T helper cells (CD3^+^CD4^+^CD8^-^), memory T-helper cells (CD3^+^CD4^+^CD8^+^) and naïve T cells (CD3^+^CD4^-^CD8^-^). In addition, we quantified the total proportion of CD3^+^CD4^+^, CD3^+^CD8^+^ and the ratio of CD4^+^ to CD8^+^ (CD4^+^/CD8^+^).

### 2.4 Statistics of PBMCs subpopulations

Basic descriptive statistics of absolute counts and proportion of the different analysed lymphocyte subsets of cells among PBMCs are shown in **Table 1**. Normality of each PBMC subpopulation data was checked through Shapiro-Wilk test in R. For some of the variables, logarithm or square root transformations were applied to reach normal distribution of residuals (p-value > 0.05).

**Table 1:** Descriptive statistics of absolute numbers and proportions of peripheral blood mononuclear cells (PBMCs).

Trait	Cell counts			% of PBMCs		
	Mean	SD	CV	Mean	SD	CV
T lymphocytes	10327.01	2466.48	0.24	43.81	9.19	0.21
CTL cells	4563.98	2039.04	0.45	19.41	8.52	0.44
Memory T cells	748.85	671.54	0.90	3.18	2.79	0.88
T helper cells	428.55	216.16	0.50	1.84	0.95	0.52
Naïve T cells	4585.62	2035.98	0.44	19.37	8.23	0.43
Natural killer (NK) cells	3346.56	1479.30	0.44	14.16	6.12	0.43
B lymphocytes	5048.28	1938.44	0.38	21.22	7.84	0.37
CD4^+^ T cells	1177.41	776.91	0.66	5.02	3.25	0.65
CD8^+^ T cells	5312.84	2294.21	0.43	22.59	9.49	0.42
CD4^+^/CD8^+^	0.12	0.09	0.78	–	–	–

standard deviation (SD), coefficient of variation (CV).

A linear regression model was then applied on transformed and raw data using the *lm()* function in R with sex and batch as fixed effects to test their significance on each cell subset through likelihood ratio tests. When significant, sex and/or batch effects were considered for subsequent analyses.

Pairwise phenotypic correlations (rp) among all analysed phenotypes were computed after adjusting for significant systematic factors.

### 2.5 Estimation of genetic parameters

The heritability (*h* ^2^) , i.e. the proportion of phenotypic variance attributable to additive genetic effects, was estimated for the percentage of the different lymphocyte subsets of cells showed in **Table 1**. Variance components and the corresponding *h^2^* were estimated from an univariate animal model as follows:

***Y=Xβ+Zu+e***

where ***Y*** is the vector of phenotypes, the percentage of lymphocyte subpopulations (either transformed or raw data) of all individuals; ***β*** is the vector of systematic (fixed) effects on the trait, including when significant the effects of sex (2 levels) and/or batch (6 levels); ***u*** is the vector of animal’s genetic additive (random) effects; ***X*** and ***Z*** are the corresponding incidence matrices for ***β*** and ***u*** ; and ***e*** is the vector of random residual terms. The assumed distribution of additive genetic effects was **u**∼(0, **A** $\sigma_{u}^{2}$ ), where **A** is the numerator relationship matrix computed on the basis of pedigree and $\sigma_{u}^{2}$ is the additive genetic variance; random errors were distributed as e∼*N*(0,I $\sigma_{e}^{2}$ ). Estimation of the model variance components and the corresponding heritability $\left(h^{2}=\;\sigma_{u}^{2}/\left(\sigma_{u}^{2}+\sigma_{e}^{2}\right)\right)$ for each lymphocytes subset was performed by REML using the aireml program included in the BGF90 package (17). The standard errors (SE) of the heritability estimates were also computed, thus obtaining the corresponding confidence intervals at 95% (CI95).

Subsequently, pairwise genetic correlations (for each two traits combination) were estimated in a two-traits animal model described as follows:


$$\left[\begin{array}{c} Y_{t1}\\ Y_{t2} \end{array}\right]=\left[\begin{array}{cc} X_{t1} & 0\\ 0 & X_{t2} \end{array}\right]\left[\begin{array}{c} \beta_{t1}\\ \beta_{t2} \end{array}\right]+\left[\begin{array}{cc} Z_{t1} & 0\\ 0 & Z_{t2} \end{array}\right]\left[\begin{array}{c} u_{t1}\\ u_{t2} \end{array}\right]+\left[\begin{array}{c} e_{t1}\\ e_{t2} \end{array}\right]$$

where ***Y***_*t*1_  and ***Y***_*t*2_ are the vectors of phenotypic observations for trait 1 and trait 2, respectively; ***β***_*t*1_ and ***β***_*t*2_ are the vectors of systematic (fixed) effects on each trait previously described, and ***X***_*t*1_ and ***X***_*t*2_ the correspondent incidence matrices; ***u***_*t*1_ and ***u***_*t*2_ are the vectors of animal genetic additive effects on trait 1 or trait 2 (random effects), and ***Z***_*t*1_ and ***Z***_*t*2_ the corresponding incidence matrices; finally ***e***_*t*1_ and ***e***_*t*2_ are the vectors of residual errors for each trait. The (co)variance matrix of random genetic effects was defined as:


$$Var\left[\begin{array}{c} u_{t1}\\ u_{t2} \end{array}\right]=\left[\begin{array}{cc} A\sigma_{u1}^{2} & A\sigma_{u1,u2}\\ A\sigma_{u1,u2} & A\sigma_{u1}^{2} \end{array}\right]$$

where $\;\sigma_{u1}^{2}$ and $\sigma_{u2}^{2}$ are the additive genetic variance of traits 1 and 2, respectively, *σ*_***u*** 1, ***u*** 2_ is the genetic covariance between the traits, and **A** is the numerator relationship matrix as defined above. Estimation of the (co)variance components of each pairwise analysis was also performed by REML using the aireml program included in the BGF90 package (17). Genetic correlations (rg) between traits were obtained as rg= (σu1,u2σu1σu2) , and the SE of the genetic correlation estimates were also computed.

### 2.6 SNP genotyping

The GGP Porcine HD Array (Illumina, San Diego, CA) was employed to genotype 68,516 single nucleotide polymorphisms (SNPs) in the 391 animals using the Infinium HD Assay Ultra protocol (Illumina). Plink software (18) was used to remove those SNPs with a minor allele frequency (MAF) lesser than 5%, SNPs with more than 10% missing genotypes, and SNPs that did not map to the porcine reference genome (*Sscrofa11.1* assembly). Finally, a subset of 42,641 SNPs remained for further analysis.

### 2.7 Genome wide association study (GWAS)

GWAS was carried out between the 42,641 filtered SNPs and the 10 traits related to the percentages of PBMCs subpopulations, described in **Table 1**. To this end, the genome-wide complex trait analysis (GCTA) software (19) was used following an additive model for each trait across all SNPs:


$$Y=X\beta +Zg+S_{l}\;a_{l}+e$$

where ***Y*** corresponds to the vector of phenotypic observations (log or sqrt transformed or raw data) for each analysed trait; ***β*** is the vector of systematic effects on the trait described above; ***g*** is the vector of infinitesimal genetic effects of each individual, with distribution ***g*** ∼*N*(0,**G** $\sigma_{g}^{2}$ ), where **G** is the genomic relationship matrix (GRM) calculated using the filtered autosomal SNPs based on the methodology of (19) and $\sigma_{g}^{2}$ is the additive genetic variance; ***S*_*l*_** is the vector of genotypes (coded as 0, 1, 2) of each individual for the *l^th^* SNP, and *a*_*l*_ is the allele substitution effect of the *l^th^* SNP on the trait under study. The FDR method of multiple testing described by Benjamini and Hochberg (20) was used to measure the statistical significance for association studies at genome-wide level with the p.adjust function of R. The significant association threshold was set at FDR ≤ 0.1. Manhattan plots based on the significance of the associations across the whole genome were generated using the qqman R package (21). Quantile-quantile (Q-Q) plots of the p-value distribution were generated with the CMplot R package available at: https://github.com/YinLiLin/CMplot.

VEP software (22) was used to locate all the significant SNPs and determine their functional effects. Gene annotation for 1Mb downstream/upstream of genomic intervals around the most significant SNPs was performed with BIOMART software (23) using the Ensembl Genes 106 Database of Scrofa11.1 reference assembly. Gene features and gene ontologies (GO) were retrieved. For those pig genes without a name, orthologous human gene names were retrieved. Furthermore, information from Genecards (24) and Mouse Genome Database (25) was used to identify gene functions affecting the analysed phenotypes.

### 2.8 RNA-Seq of blood

RNA-Seq of whole blood was performed for 30 pigs classified as low (L) or high (H) percentage of T γδ (L=12, H=12), T helper and memory T-helper cells (L=15, H=15), which were selected from the 391 pigs of the Duroc population by a principal component analysis (PCA; **Figure S2**). PC2 was considered to classify 30 animals extreme for T helper (H group: 3.49 ± 0.25; L group: 0.76 ± 0.06) and memory T helper cells (H group: 9.92 ± 1.16; L group: 1 ± 0.18). Since these two populations showed the same direction in the PCA, we considered the same animals per groups. Subsequently, these 30 animals were also classified for extreme proportions of T γδ cells considering the phenotype by (12), where PBMCs were stained with a specific antibody for T γδ cells (APC Rat Anti-Pig γδ T Lymphocytes, clone MAC320, BD Biosciences). For this analysis, we selected 12 animals per group to maximize the differences in the proportions of T γδ cells (H group: 16.36 ± 1.86; L group: 3.64 ± 0.51).

Total RNA from blood was isolated and purified with a Tempus™ Spin RNA Isolation Kit (Thermo Fisher Scientific, Spain), following the manufacturer’s instructions. Total RNA concentration was measured in a Nanodrop One spectrophotometer (Thermo Fisher Scientific, Spain) and RNA purity and integrity was checked by using a Fragment Analyzer equipment (Agilent Technologies, INC., Santa Clara, CA). Libraries were prepared using a Stranded total RNA Prep with Ribo-Zero Plus kit (Illumina Inc., CA) and were paired-end sequenced (2 × 100 bp) on a NovaSeq 6000 platform (Illumina Inc., CA) at the National Centre for Genomic Analysis (CNAG, Barcelona, Spain). More than 50 million of paired-end reads were obtained for all samples. The quality of the raw sequenced reads in the FASTQ files was analysed with the FASTQC software (Babraham Bioinformatics, http://www.bioinformatics.babraham.ac.uk/projects/fastqc/). Reads were mapped to the reference pig genome Sscrofa11.1 and the annotation database Ensembl Genes 106 by using STAR v. 2.75.3a. Transcript quantification was performed with RSEM v. 1.3.0. The EdgeR R package (26) was used to identify differentially expressed (DE) genes between the two divergent groups (L vs H). After correcting for multiple testing, genes with a Fold Change (FC) between groups greater than 1.2 (i.e. |log2FC| > 0.26) and an FDR< 0.1 were classified as DE. Enriched GO terms and pathway analysis (padj<0.1) of the DE genes was performed with the ClueGO v2.5.8 plug-in of Cytoscape v3.9.1 (27).

## 3 Results

### 3.1 Characterization of immune phenotypes

In the present study, we stained PBMCs to measure ten immune-cell-related traits including the relative abundance of different populations of lymphocytes, the proportions of CD4^+^ T cells and CD8^+^ T cells, and the ratio of CD4^+^/CD8^+^ T cells from 391 individuals of a commercial Duroc pig line. All proportions and absolute numbers of cell subsets with descriptive statistics are reported in **Table 1**. The T cells were the most abundant population of lymphocytes, representing 43.8% of PBMCs. Among T lymphocytes subpopulations, the CTL and naïve T cells were the most abundant (~19% of PBMCs each one), whereas the memory plus helper T cells (*i.e.* CD4^+^ T cells) represented 5% of PBMCs. The mean proportion of B lymphocytes (21.2% of PBMCs) was less than half that of T lymphocytes, whereas the NK cells represented 14.16% of PBMCs. A high variability between individuals was observed in the relative abundance of some PBMC types, the CV ranging from 0.21 to 0.89. The percentage of T lymphocytes showed the lowest dispersion (CV=0.21), although within T cells, the percentage of memory T cells showed the highest variability (CV=0.88) among the analysed PBMCs subtypes.

Phenotypic correlations were also computed to analyse the association between the relative abundance of the different lymphocyte subsets (**Supplementary Table 2**). Positive correlations were observed between proportions of T cells and its different subpopulations, ranging from 0.34 to 0.51, but for T helper cells, that exhibited a low correlation with T cells percentage. Among T cells subtypes, the relative abundance of memory T-helper cells correlated positively with both T-helper and CTL cells (r_P_= 0.39 and 0.32), whereas an antagonism between the proportions of CTL and naïve T cells was observed (r_P_= -0.50). The relative abundance of B lymphocytes showed a strong negative correlation with total T lymphocytes and CTL proportions of PBMCs (r_P_= -0.56 and -0.50). NK cells proportion was positively correlated to CTL abundance but showed negative correlations with the relative abundances of naïve T cells and B lymphocytes (r_P_= -0.51 and -0.45, respectively). As expected, the proportion of CD4^+^ T cells was highly correlated with T helper and memory T-helper cells. The proportion of CD8^+^ T cells was highly correlated with CTL T cells and moderately correlated with T cells abundance, whereas it showed antagonism with Naïve T cells and B lymphocytes proportions.

Additionally, phenotypic correlations of the different lymphocytes subtypes abundance with a plethora of innate immunity traits previously analysed in the same individuals (12), including plasma immunoglobulins and acute phase proteins concentrations, γδ T lymphocytes, haemogram figures and phagocytosis capacity, were also computed (**Supplementary Table 2**). Among these traits, only the γδ T lymphocytes showed a strong and highly significant phenotypic association with naïve T cells (r_p_ = 0.81, p-value = 2.2×10^-16^), indicating that naïve T cells (CD3^+^ CD4^-^ CD8^-^) corresponded mainly to γδ T cells. The γδ T cells proportion also showed a moderate correlation with T cells and certain antagonism with NK and CTL cells proportions. Finally, the percentage of phagocytic cells and the phagocytosis capacity of lymphocytes showed low but significant positive associations with the percentage of B cells, jointly with low negative correlations with T cells (CTL and Memory T cells) and NK relative abundances.

### 3.2 Genetic parameters of T and B-cell populations

The heritability of the different cell immunity subsets was estimated to ascertain the genetic determinism of cellular immunocompetence traits. Medium to high heritabilities were observed for the ten immune-cell-related traits comprising the relative abundance among PBMCs of different lymphocyte populations (**Table 2**). The heritability estimates ranged between 0.361 to 0.841, being the proportion of memory T-helper cells (h^2 =^ 0.841) the most heritable trait, followed by the total proportion of T cells (h^2 =^ 0.771). In contrast, the percentage of B cells exhibited the lowest heritability value (h^2 =^ 0.361); despite moderate, the 95% confidence interval for its heritability did not encompasses zero (**Table 2**). The NK cells abundance and the proportion of different T cells subtypes except for memory T-helper cells showed medium heritabilities between 0.470 and 0.558.

**Table 2:** Heritability estimates (*h^2^*) for the relative abundance (among PBMCs) of different cell immunity subsets, plus standard errors (SE) and confidence intervals at 95% (CI95) of the *h^2^* estimates.

Trait	h^2^	SE	CI95
T lymphocytes (% of PBMCs)	0.771	0.152	0.473 - 1.070
CTL cells (% of PBMCs)	0.563	0.155	0.259 - 0.868
Memory T cells (% of PBMCs)	0.841	0.161	0.526 - 1.157
T helper cells (% of PBMCs)	0.470	0.148	0.181 - 0.759
Naïve T cells (% of PBMCs)	0.481	0.144	0.198 - 0.764
B lymphocytes (% of PBMCs)	0.361	0.165	0.036 - 0.685
Natural killer cells (% of PBMCs)	0.558	0.168	0.228 - 0.887
CD4^+^ T cells (% of PBMCs)	0.725	0.159	0.413 - 1.036
CD8^+^ T cells (% of PBMCs)	0.487	0.158	0.178 - 0.796
CD4^+^/CD8^+^	0.462	0.141	0.186 - 0.738

**Figure 1:**
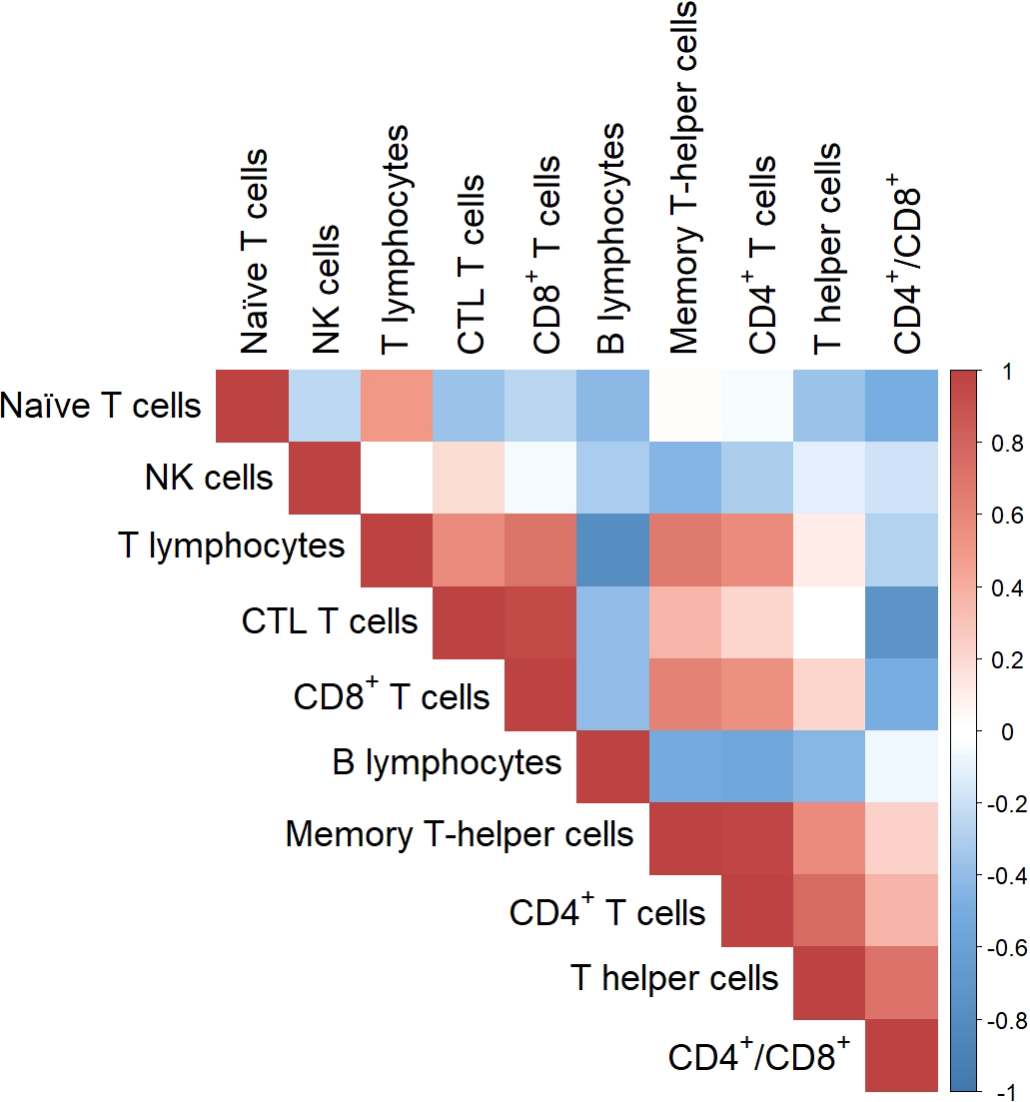
Heatmap of genetic correlations estimated by pairwise combination among cell immunity traits.

When we inferred the genetic correlations between each pairwise combination of traits, positive and negative genetic associations were identified between the proportion (among PBMCs) of some lymphocytes populations (**Figure 1** and **Supplementary Table 3**). In general the genetic correlation pattern matched with that observed with phenotypic correlations, but stronger associations were observed at genetic level despite its significance being limited in a number of cases due to the sample size of the population. Positive and significant genetic correlations were obtained between the proportion of T cells and the different T cells subpopulations (r_g_ from 0.50 to 0.68) with the exception of T helper cells. Among T cells subpopulations, a positive genetic association of memory T-helper cells with T helper (r_g_ = 0.58) and CTL (r_g_ = 0.37) cells was estimated, whereas naïve T cells were negatively correlated with CTL and T helper cells (r_g_ = -0.37 and -0.36). The proportion of B lymphocytes exhibited negative genetic correlations with the rest of lymphocytes subsets, particularly with the total percentage of T cells (r_g_ = -0.78), CD4^+^ T cells (r_g_ = -0.56) and CTL T cells (r_g_ = -0.41). NK cells proportion were negatively correlated with memory T-helper cells (r_g_ = -0.44) and to a lesser extent with B lymphocytes (r_g_ = -0.32) proportion.

As far as genetic correlation of the analysed PBMCs subsets with innate immunity and other health-related traits (**Supplementary Table 3**), we identified a strong genetic correlation between the proportion of γδ T lymphocytes and naïve T cells (r_g_ = 0.903) subpopulation, which corroborates that CD3^+^ CD4^-^ CD8^-^ T cells were mainly γδ T cells, jointly with a genetic antagonism between the proportion of γδ T and T helper lymphocytes subpopulation (r_g_ = -0.568). The CTL abundance showed positive genetic correlation with the number of lymphocytes and other white blood cell counts in the haemogram (r_g_ from 0.622 to 0.702) as well as with haematocrit (r_g_ = 0.741). Besides, negative genetic correlations of the proportion of B lymphocytes with both the phagocytic capacity and the percentage of phagocytic cells (r_g_ = -0.803 and -0.733, respectively) were obtained, in contrast with the positive correlation with the percentage of phagocytic lymphocytes (r_g_ = 0.544).

### 3.3 Genomic regions and candidate genes associated with immune-cell traits

To identify genomic regions associated with lymphocytic traits, a GWAS was performed using the ten immune-cell-related traits and the genotypes of 42,641 SNPs of the Porcine GGPSNP70 BeadChip (Illumina) in 391 Duroc pigs. Significant associations at whole-genome level (FDR ≤ 0.1) were detected for T helper cells, memory T-helper cells and naïve T cells (**Table 3**). A total of 32 significantly associated SNPs located at four chromosomal regions on pig chromosomes SSC3, SSC5, SSC8 and SSCX were identified (**Supplementary Table 4**). All the associated SNPs, with their predicted consequences, are shown in **Supplementary Table 5**. In addition, the identified genomic region on SSC5 was also associated with the total proportion of CD4^+^ T cells at FDR threshold< 0.2 (**Table 3** and **Figure S3**).

**Table 3:** Description of the four chromosomal regions associated with cell immunity traits and the annotated candidate genes.

Region	Chr	Start (Mbp)	End (Mbp)	N SNPs	Top SNPs	MAF	p-value	FDR	Trait	Candidate genes
1	3	55.60	58.34	14	rs81340900, rs81293514, rs81336780	0.49	2.18E-06	3.10E-02	T helper cells (% of PBMCs)	*CD8A, CD8B, CHMP3, GNLY, IGKV, RMND5A, SMYD1, ZAP70*
2	5	61.62	62.44	10	rs326461238	0.26	1.18E-05	8.70E-02	Memory T cells (% of PBMCs) CD4^+^ T cells	*AICDA, CD4, CD69*, *CD163, MFAP5, PHC1, STYK1, TAS2Rs, KLRs, C-type lectins*
3	8	20.51	20.57	2	rs81406759, rs81406807	0.40	3.45E-05	9.80E-02	T helper cells (% of PBMCs)	*RBPJ, STIM2*
4	X	33.36	33.63	6	rs342772739	0.13	1.18E-06	1.20E-02	Naïve T cells (% of PBMCs)	*CYBB, ssc-mir-9786-1*

**Figure 2:**
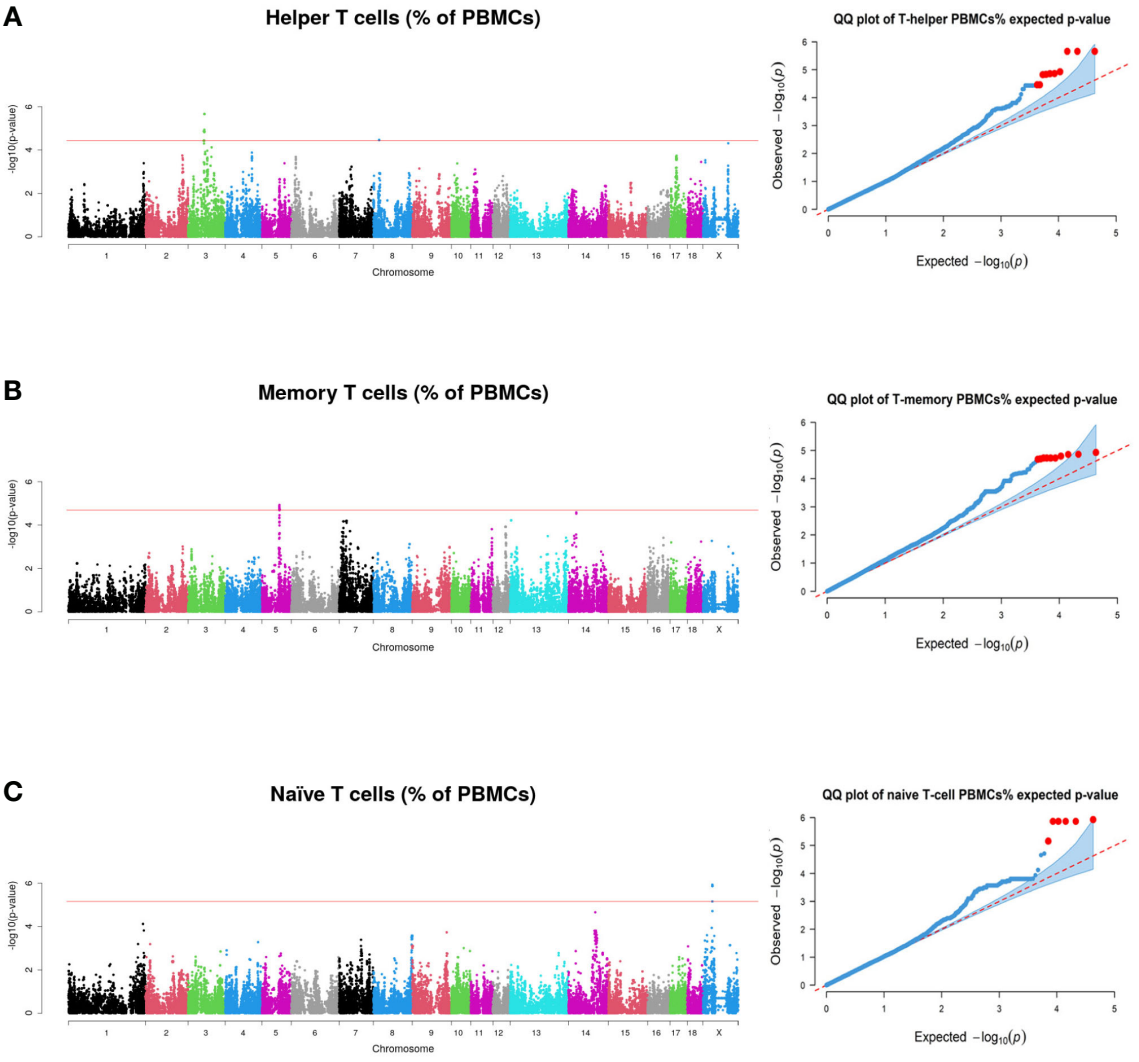
Manhattan plots and quantile-quantile plots representing the p-values profiles corresponding to the association analysis between immunity traits and SNPs, **(A)** for Helper T cells **(B)** for Memory T cells and **(C)** for Naïve T cells. Red line indicates those SNPs that are below the genome-wide significance threshold (FDR ≤ 0.1).

Graphical representation displayed in Manhattan plots of the GWAS results for T helper (A), memory T-helper (B) and naïve T (C) cells are shown in **Figure 2**. Furthermore, Q-Q plots of the data represented in the Manhattan plots are also shown in **Figure 2**.

In SSC3, a region comprising 55.60 to 58.34 Mb was declared to be associated with the proportion of T helper cells (**Figure 2A**). Genes related to immune response were found annotated in this region: the family of immunoglobulin kappa variable region genes, T-cell surface glycoproteins (*CD8A* and *CD8B*), Required for Meiotic Nuclear Division 5 Homolog A (*RMND5A*), Charged Multivesicular Body Protein 3 (*CHMP3*), SET and MYND Domain Containing 1 (*SMYD1*), Zeta Chain of T cell Receptor Associated Protein Kinase 70 (*ZAP70*) and Granulysin (*GNLY*). The three top significant associated SNPs (rs81340900, rs81293514 and rs81336780) were located in an intron of the *RMND5A* gene. In addition, two significant SNPs at 20.51-20.57 Mb of SSC8 were also associated with the variation of T helper cells (**Figure 2A** and **Table 3**). In this region, Recombination Signal Binding Protein for Immunoglobulin Kappa J region (*RBPJ*) and Stromal Interaction Molecule 2 (*STIM2*) genes were annotated.

In SSC5, 10 significant SNPs at 61.97 to 62.29 Mb were associated with the proportion of memory T-helper cells (**Figure 2B**), being rs326461238 the most significant one (**Table 2**). In this region, a high number of immune-related genes (*AICDA*, *CD4*, *CD69*, *CD163*, *MFAP5*, *PHC1*, *STYK1*, a family of TAS2Rs, a family of KLR such as *KLRB1*, *KLRC1*, *KLRK1* and *KLRD1*, and a family of C-type lectins such as *CLECL1*, *CLEC4D* and *CLEC4E*) were identified. Furthermore, the most associated SNP was found inside a long-noncoding RNA (lncRNA, LOC100524679).

Finally, six SNPs at 33.36 to 33.63 Mb in SSCX were associated with naïve T cells (**Figure 2C**). The most significant SNP (rs342772739) was found in the locus for *LANCL3* gene. Despite that, no reports have been found to indicate that the gene is directly associated with immunity phenotypes. In addition, *CYBB* gene, also related to immunity functions, was found in this region.

### 3.4 Comparison with other GWAS studies

To identify overlaps between our QTL regions and those previously identified in pigs and humans for immune cellular traits a comparison between genomic regions and candidate genes was performed. Remarkably, we found several candidates genes previously reported as candidates in human GWAS studies for the same or similar phenotypes such as *CD8A*, *CD8B*, *RMND5A* and *VPS24* (also known as *CHMP3)* for CD4^+^ proliferating, CD4^+^CD8^dim^ T cell Absolute Counts, CD4^+^CD8^dim^ T cell % lymphocytes and CD4^+^CD8^dim^ T cell % leukocyte traits (9, 14, 28) and *CD4* and *KLRC2* for CD4^+^CD8^+^ and effector memory CD4^+^ T cell % T cell traits (9, 28, 29). In pigs, two overlapping or very close genomic regions for T helper cells and memory T-helper cells were identified. Regarding T helper cells trait, overlapping regions for leukocyte percentages of CD8^+^, CD8^-^ and CD3^-^CD8^-^ traits of an F2 Duroc × Erhualian piglets vaccinated with a live CSF vaccine were identified (15). A region at 55 Mbp in SSC5 for CD4^+^CD8^+^ percentage of leukocytes and located less than 7 Mb away from the region identified in our analysis for CD4^+^CD8^+^ memory T-helper cells was identified in a population of animals consisting of Landrace, Yorkshire, and Songlio Black pigs (16).

### 3.5 Genes differentially expressed between groups diverging in percentage of T γδ, T helper and T memory cells

The transcriptome of whole blood samples from animals with extreme proportions of T γδ, T helper and memory T-helper cells was analysed by RNAseq to identify genes DE between groups.

**Figure 3:**
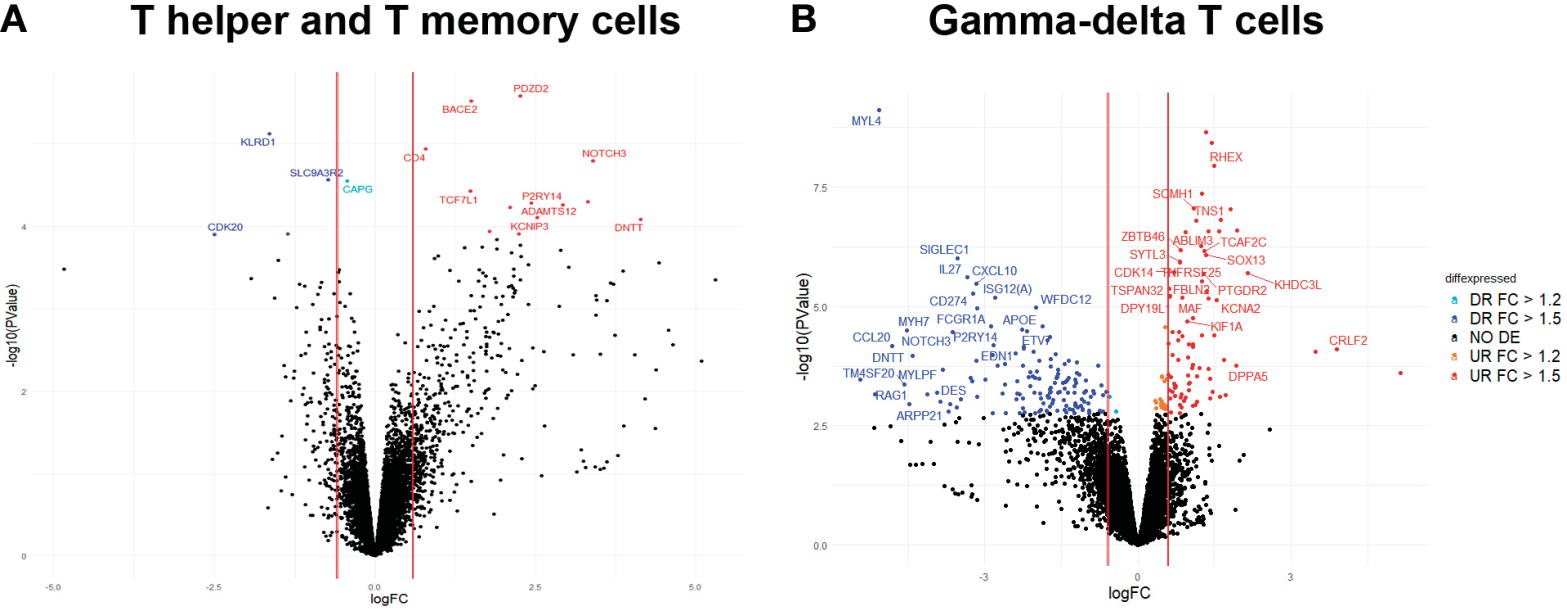
Volcano plot displaying DE genes in blood between H and L groups for **(A)** T helper and memory T-helper cell and **(B)** δγ T cells. The vertical axis (y-axis) corresponds to the -log10 (P-value), and the horizontal axis (x-axis) displays the log_2_ fold change (logFC) value. The vertical lines mark the thresholds located to FC = 1.5 and FC = -1.5. Blue dots represent downregulated genes in HvsL groups with FC< -1.5 and FDR< 0.1. Cyan dots represent downregulated genes in HvsL groups with -1.5< FC< -1.2 and FDR< 0.1. Red dots represent upregulated genes in HvsL groups with FC > 1.5 and FDR< 0.1.

A total of 18 genes were identified as DE (FC>1.2; FDR<0.1) in blood between the two groups of animals with high (H) and low (L) T helper and T memory cell percentages. From them, 5 genes were downregulated and 13 were upregulated in the H group when compared to the L group (**Figure 3A** and **Supplementary Table 6**). The most up- and downregulated genes in the H group were DNA nucleotidylexotransferase (*DNTT*; FC = 17.65, *P*-value = 8.19 x 10^-05^) and Cyclin dependent kinase 20 (*CDK20*; FC^−1^ = 5.67, *P*-value = 1.24 x 10^-04^), respectively.

The functional annotation of DE genes identified *CD4* as being involved in “helper T cell enhancement of adaptive immune response” GO immune system process, among other immune-related functions (**Supplementary Table 7**). Other genes related to immune functions were *KLRD1*, *NOTCH3* and *TCF7L1* (**Supplementary Table 7**). Furthermore, the following genes *CAPG*, *DNTT*, *KCNIP3*, and *P2RY14* have also been reported in the literature to be related to immunity (30–32).

Regarding blood transcriptomic changes between H and L groups for percentages of γδ T cells, 246 DE genes were identified (FC>1.2; FDR<0.1; **Figure 3B** and **Supplementary Table 8**). Of these, 102 genes were upregulated and 144 were downregulated in the H group when compared to the L group. The most up- and downregulated genes in the H group were the novel genes ENSSSCG00000046089 (FC = 35.25, *P*-value = 2.48 x 10^-04^) and ENSSSCG00000038561 (FC^−1^ = 43.43, *P*-value = 3.41 x 10^-04^), respectively. Out of the DE genes, 215 were identified as protein coding genes, 24 as lncRNAs, and 7 as pseudogenes.

After performing a functional analysis with the list of DE genes, we mainly identified GO immune system processes associated with α-β T cell differentiation and activation from the list of downregulated genes in H group, gathering animals with high proportion of γδ T cells, compared to L group. Other processes identified were negative regulation of lymphocyte activation and proliferation. Some genes identified in these processes were *CD274*, *HLX*, *IL27*, *LGALS9B*, *MYB*, *RORC*, *RSAD2* and *SOCS1* (**Supplementary Table 9**). Remarkably, we identified in the list of upregulated genes in H group a transcriptional factor (SOX13) associated with positive regulation of γδ T cell activation and differentiation. Immune processes also identified from the list of up-regulated genes were regulation of B cell receptor signalling pathway (*BLK* and *GCSAML*), regulation of erythrocyte (*ACVR2A*, *RHEX*), and dendritic, monocyte and macrophage (*ZBTB46*) differentiation, neutrophil-mediated functions (*DPP4*, *CTSG* and *ADAM8*) and T cell differentiation in thymus (*ADAM8*).

### 3.6 Coincident association between GWAS and DE analyses

In order to identify potential candidate genes whose differences in gene expression may be modulating our cellular phenotypic traits, we overlapped the lists of DE genes and candidate genes located in associated genomic regions for T helper, memory T-helper and γδ T cells traits. We identified four DE genes between groups of animals with extreme proportions of T helper and memory T-helper cells: *CAPG* and *TCF7L1* on SSC3, and *KLRD1* and *CD4* on SSC5, at or very close to the genomic regions associated with T helper cells and memory T-helper cell, respectively. No genes colocalizing between GWAS and DE studies were identified for δγ T cells.

## 4 Discussion

Nowadays, the pig industry is facing new challenges that make necessary to reorient breeding programs to produce more robust and resilient pig populations while maintaining production efficiency. It is known that the immune system and particularly lymphocytes play an important role in controlling many porcine pathogens. In this study, we focused our analysis on peripheral blood mononuclear cell traits, mainly related to adaptive immunity, but also including cell populations such as δγ T cells with functions associated with both arms of immunity.

After PBMC immunophenotyping, the relative abundance among PBMCs of total T and B lymphocytes in the analysed Duroc population was similar to figures reported in literature for other pig populations (33–35). However, differences with other studies were observed in the proportion of some T-cells subsets, with CD4^+^ cells showing lower relative abundance than that reported before (36). Although we could not discard an effect on immunophenotyping associated to handling and freezing processes, other factors such as the age of animals, environmental factors or the lack of standardization of laboratory tests could also have generated discrepancies between studies. It is also plausible that differences in the genetic background of the porcine populations analysed in these studies could be behind the differences in the abundance of certain lymphocytes subsets. For instance, genetic variation of *CD4* has been associated to a different reactivity to the CD4 antibody (36). In our study, *CD4* was one of the DE genes between animals with extreme phenotypes for T helper and memory T-helper cells, and was located in a genomic region associated with proportion of CD4^+^ and memory T cells, evidencing a genetic contribution to the variation of these traits in our Duroc population. Therefore, changes in CD4 mRNA levels among animals may be the most plausible explanation for the differences in CD4 T cells percentages observed between studies.

A particularly high heritability was estimated for both memory and CD4*^+^* T-cells relative abundances, whereas medium to high heritabilities were obtained for the rest of immune-cell-related traits in our Duroc population. These heritability values are in line with those obtained in previous human and pig studies, reaffirming genetics as an important determinant of adaptive cell immune traits (8, 10, 28, 37). The lowest heritability among PBMCs subpopulations relative abundances was observed for B cells. This is concordant with results in humans (29), showing that B cell counts were more environmentally influenced while T cells were more strongly driven by genetic factors.

The map of genetic correlations among PBMC subtypes relative abundances reported positive and negative genetic associations between these immune-cell-related traits, but also reflected the interdependence between some of these compositional variables and should be interpreted with caution. The antagonism between T and B cells proportions in blood observed in our Duroc population might have some physiological implications deserving further research. However, we cannot discard this antagonism to be the consequence of dealing with compositional data. Conversely, we can affirm that our results confirm a positive genetic association between memory and T-helper cells in blood composition. In this regard, two significant genomic regions associated with the proportion of T helper and memory T-helper cells in blood were identified. This may allow selection of animals to investigate abundance of these cells in blood in relation to immune responses toward different stimuli.

Selecting animals for producing higher immune responses should probably consider both innate and adaptive immunity traits in conjunction with qualitative responses. The map of genetic correlations of PBMCs subpopulation proportions with innate immunity traits might help to evaluate the consequences/convenience of selecting for both innate and adaptive immunity traits. Most PBMCs subset abundances (but naïve T cells) showed a negligible genetic association with the concentration of acute phase proteins (CRP and Hp) in blood. Similar results were shown by (10), whereas a positive genetic correlation between the concentration of CRP and B cell counts was described by (38) in animals tested at 90kg of live weight. The genetic independence of acute phase proteins concentration versus most lymphocytes subsets abundance observed in our Duroc population would support the possibility of selecting some immune-cell-related traits independently of some innate immune pathways governing CRP synthesis and then avoiding adverse effects due to inflammation.

Genetic correlations of the different PBMC subpopulations proportions with other immunity traits also pinpoint to other interesting results. The relative abundance of CTL T cells was positively associated at the genetic level with haematological traits, particularly with leucocyte counts and with the haematocrit. Conversely, our results also suggest certain genetic antagonism between the proportion of B lymphocytes and the phagocytic capacity, and between some T cells subpopulations (particularly CTL and memory T-cells) and serum immunoglobulin concentrations. As a whole, the genetic interaction map among innate and adaptive immunity traits obtained in our Duroc population partially support the possibility of applying an effective multi-trait selection for both innate and adaptive immunity. In this scenario of complex genetic relationships, the possibility of identifying genetic markers, biomarkers and candidate genes associated to the phenotypic variation that could allow a more directed selection of some of these traits, without impairing other elements of the immune systems, takes particular relevance.

A genetic correlation close to unity was observed between naïve and δγ T cells quantified with a specific monoclonal antibody (MAC320) in a previous study in the same Duroc population (12), showing that most of the CD4^-^CD8^-^ T cells were in fact γδ T lymphocytes. Consequently, GWAS study identified the same genomic region on SSCX using both phenotypic datasets (12). In the present study, we annotated a new promising candidate gene, *CYBB* encoding for one of the two chains of cytochrome b (CYB). CYB is a component of the microbicidal oxidase system expressed by neutrophils and T cells, a critical process of innate immunity for defence against bacterial and fungal infections. Mutations in human *CYBB* have been previously associated to immune system dysfunction and can lead to chronic granulomatous disease (39). Since δγ T cells constitute a high proportion of lymphocytes in porcine peripheral blood and could have the potential to combine conventional adaptive and innate-like responses (35, 40), the activation of WC1-bearing δγ T cells has emerged as a potential alternative to optimize new vaccine strategies (41). In this sense, an increased recruitment of δγ T cells when animals are vaccinated earlier in life appears to correlate with improved efficacy of current vaccines (reviewed in (42)). Indeed, TRDC-knockout pigs, defective in δγ T cells, showed lower neutralizing antibody titres compared to syngenic controls when they were vaccinated against classical swine fever virus using a highly attenuated live virus (43). Thus, our results indicate the possibility to select animals for higher δγ T cells counts in blood to check for potential enhancement of vaccine efficacy.

Another promising candidate genes were located in the SSC3, SSC5 and SSC8 genomic regions associated with the variation of T helper and memory T-helper cells and proportion of CD4^+^ cells. *CD8A*, *CD8B*, *GNLY* and *RBPJ* were some of the genes associated with the proportion of T helper cells. CD8a and CD8b are integral membrane glycoproteins found on the surface of many immune-cell types and act as co-receptors during antigen recognition by TCR (44). *GNLY* is an antimicrobial secretion protein present in CTLs and NK cells (45). It also functions as an alarmin, activating dendritic cells and promoting antigen-specific immune responses by using TLR4 (46). Finally, *RBPJ* encodes a transcription factor acting through the NOTCH signalling pathway to protect T-helper cells from apoptosis (47). Also associated with the percentage of memory T-helper and CD4^+^ cells we identified *CD4* and *CD69*. While CD4 is the main membrane marker for helper (naïve and memory) T-cells, it serves as a co-receptor of the TCR, enhancing recognition of the MHC class II complex (48). Of interest CD69 expressed in resident memory T cells and γδ T cells, is an activation marker determining migration-retention of T cells from tissues (49). Furthermore, we also identified *CLECL1* and the killer cell lectin-like receptors genes *KLRC1* and *KLRD1* which are highly expressed in memory T cells and are involved in their activation (*CLECL1*) and killing function (KLRs expressed by human CD8^+^ T effector memory cells) (50). Indeed, a particularly remarkable result arising in this study was the overlap of genes and genomic regions for the same or similar lymphocyte-related phenotypes with previous studies reported in pig and humans (9, 14–16, 28, 29). To the best of our knowledge, this is the first study to report overlapping between humans and pig genes (*CD4*, *CD8A*, *CD8B*, *KLRC2, RMND5A* and *VPS24*) associated with the variation of T-cell populations, further supporting the use of pig as a very reliable biomodel for human infectious diseases, vaccine development and T-cell research (51–53). Also, another remarkable result obtained in this study was the overlap between GWAS and DE analyses, pointing out genes (*CAPG*, *TCF7L1*, *KLRD1* and *CD4*) that may be modulating T lymphocytes traits through variation of its gene expression. Since transcriptional and post-transcriptional regulation of immune gene expression is critical for modulating immune responses (54, 55), eGWAS studies are required to identify potential cis and/or trans regulatory factors regulating the expression of these genes. However, we cannot rule out that the differences in the expression of some of these genes may be related to the different proportions of these populations in blood. For example, *CAPG* is a gene mainly expressed in monocytes, B cells and dendritic cells (Human Protein Atlas proteinatlas.org (56),), and was downregulated in the group with higher proportions of T helper and T memory cells. Furthermore, no overlapped genes were identified between GWAS and DE analyses for δγ T cells. However, we identified an interesting pattern of gene expression with genes related to immune processes of αβ T cell differentiation and activation downregulated in the group with higher proportion of γδ T cells. This result is in agreement with the upregulation of *SOX13* in animals with higher proportion of γδ T cells as this gene encodes a transcription factor essential for normal γδ T cell development (57). It has been reported a higher expression of *SOX13* in bovine WC1^+^ γδ T cells relative to WC1^-^ γδ T cells (58). Remarkably, we also identified three upregulated genes ENSSSCG00000034914 (CD163L1), ENSSSCG00000039217 (cow WC1 orthologue) and ENSSSCG00000031085 (antigen WC1.1) in animals with higher proportions of γδ T cells, consistent with the transcription of WC1 co-receptors by circulating γδ T cells (42). WC1^+^ cells can be divided in different subpopulations with different ability to respond to particular pathogens and cytokine responses (42). A model in cows has been proposed in which a differential occupancy on these WC1 gene loci by SOX13 influence the development of these subpopulations (58). However, still there is a lack of information about these subpopulations in swine (59). Thus, further analysis is required to determine if pigs used in the present report have different proportions of WC1^+^ subpopulations. Besides, it should be noted that the proportion of some lymphocyte subsets may vary with the age of animals (33, 34, 36), so it would be worthy to study the evolution of these lymphocytes traits throughout the life of the animals.

In conclusion this study confirmed the genetic determinism of lymphocytes traits in pigs and provide a list of promising genes and biomarkers associated to the variation of these traits. The genetic correlations described for innate and adaptive traits and the molecular markers identified for T helper, memory T-helper and γδ T cells support the possibility of applying multi-trait selection for immunocompetence in pigs. Finally, the overlap between genes for the same T cell traits in humans and pigs reinforces the use of pig as a biomodel for vaccine development and the study of human infectious diseases. Overall, our results provide new knowledge about the molecular mechanisms underlying lymphocyte traits with special interest to WC1^+^ γδ T cells to explore further optimization of current and new vaccines.

## Data availability statement

The original contributions presented in the study are included in the article/**Supplementary Material**. Further inquiries can be directed to the corresponding authors.

## Ethics statement

All experimental procedures with pigs were performed according to the Spanish Policy for Animal Protection RD 53/2013, which meets the European Union Directive 2010/63/EU about the protection of animals used in experimentation. The experimental protocol was approved by the Ethical Committee of the Institut de Recerca i Tecnologia Agroalimentàries (IRTA).

## Author contributions

MB designed the study. MB supervised the generation of the material animal used in this work. MB, OG-R, RQ and YR-C performed the sampling. AP, MB, SL-S and TJ-J carried out the laboratory analyses. AP, CH-B, DC-P, RQ, SL-S, TJ-J, YR-C and MB analysed the data and interpreted the results. MB and RQ wrote the manuscript. All authors contributed to the article and approved the submitted version.
