# Supplementary material for: Genetic architecture of innate and adaptive immune cells in pigs
Source: Front Immunol. 2023 Feb 6;14:1058346. doi: 10.3389/fimmu.2023.1058346 (PMC9939681; doi:10.3389/fimmu.2023.1058346)
Supplement: Supplementary file 1 [file DataSheet_1.docx]

Supplementary Material

# Supplementary Figures and Tables

## Supplementary Figures

**Supplementary Figure 1.** Flow cytometry gating strategy used to calculate percentages of lymphocyte subpopulations depending on PBMCs gate (A). Live-or-dyeTM Aqua viability staining (B) helped to differentiate alive (C) from dead cells. Within alive cells, CD3 (PE-CyTM7) and CD21 (PE) labelling allowed to gate NK cells (CD3- CD4- CD8+ CD21-) (D), B cells (CD3- CD21+) and T cells (CD3+ CD21-). Following T cell subsets were analysed after CD4 (Alexa Fluor® 647) and CD8 (FITC) staining into: Naïve T cells CD4- CD8-, T helper cells CD4+ CD8-, Memory T cells CD4+ CD8+ and Cytotoxic T cells (CTL) (CD4- CD8+) (E).


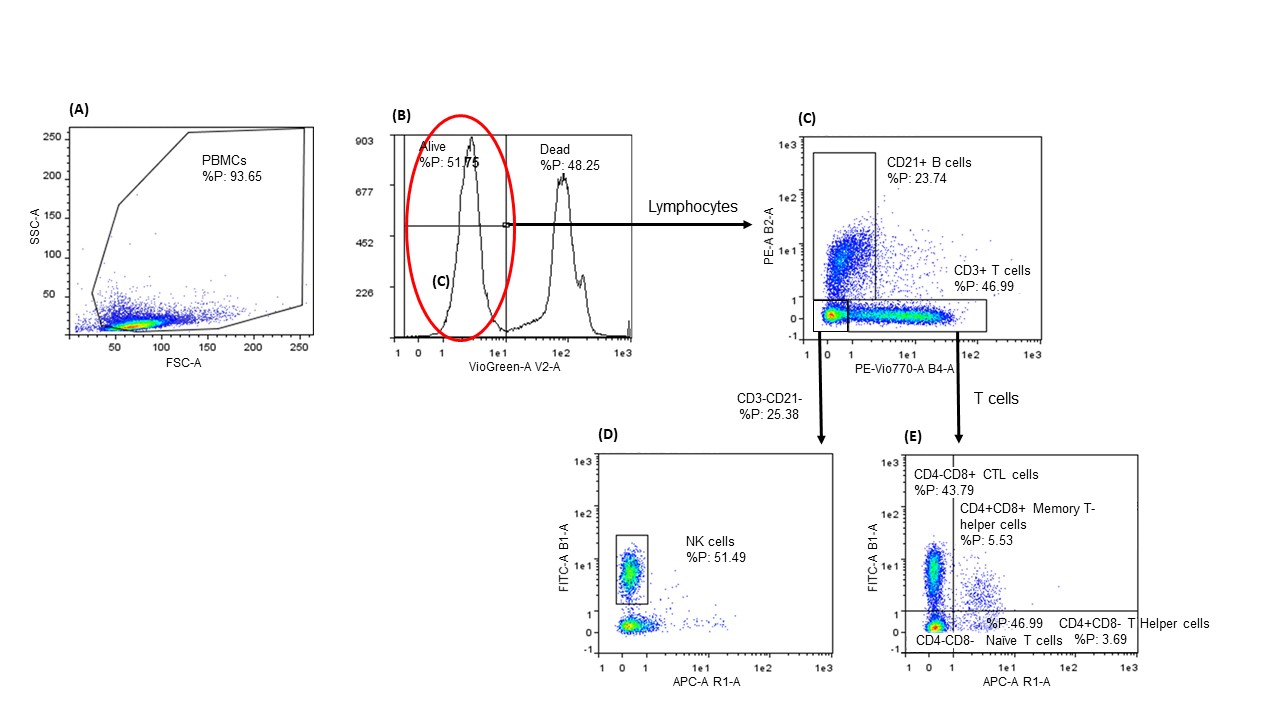


**Supplementary Figure 2.** Graphical representation of the first and second principal components summarizing the phenotype variation of traits related to immune cellular traits.


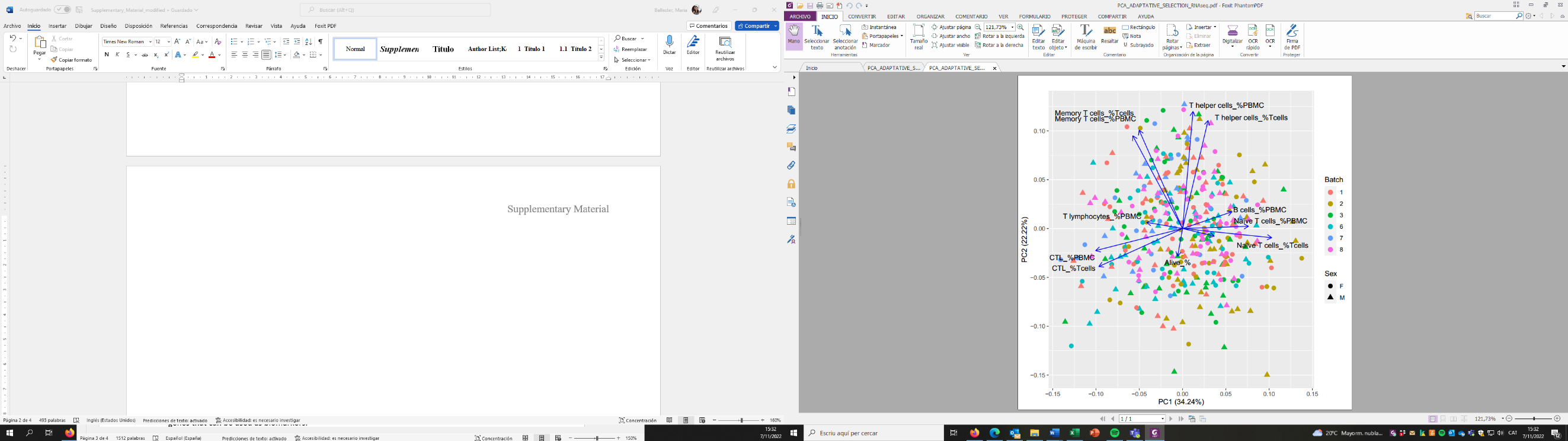


**Supplementary Figure 3.** Manhattan plot representing the association analysis between the total proportion of CD4+ T cells trait and SNPs distributed along the pig genome. Blue line indicates those SNPs that are below the genome-wide significance threshold (FDR<0.2).


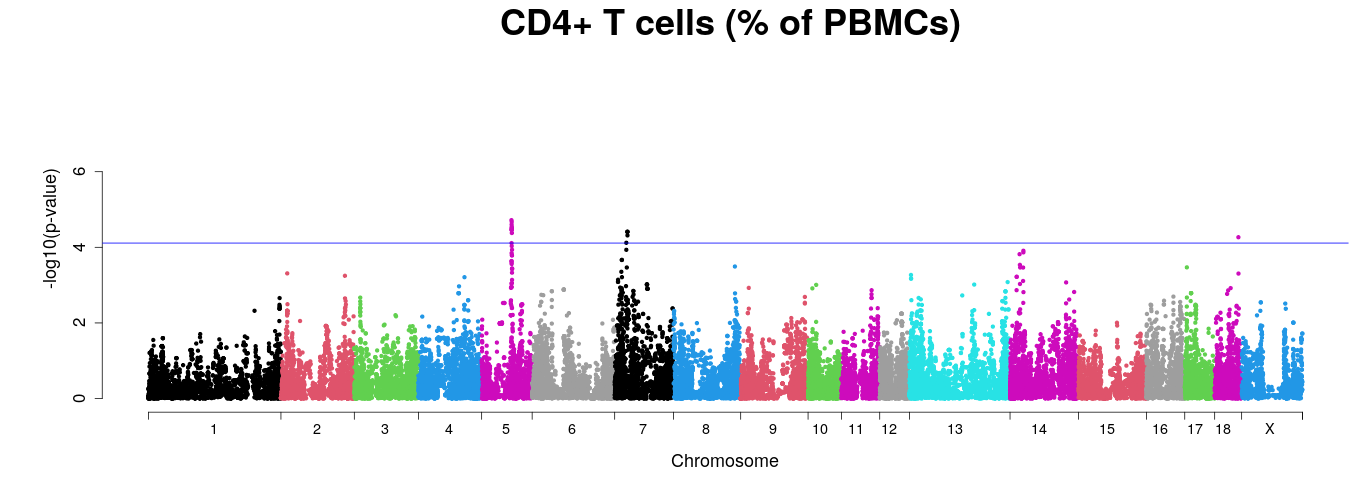


## Supplementary Tables

**Supplementary Table 1.** Antibodies used in this study*.*

| Specificities | Clone | Isotypes | Target species | Fluorochrome | Labelling strategy | Providers | Working dilutions |
| --- | --- | --- | --- | --- | --- | --- | --- |
| CD4α | 74-12-4 | Mouse IgG2b | Chick, pig | Alexa Fluor® 647 | Directly conjugated | BD biosciences | 1/200 |
| CD8α | 76-2-11 | Mouse IgG2a | Pig | FITC | Directly conjugated | BD biosciences | 1/100 |
| CD3ε | BB23-8E6-8c8 | Mouse IgG2a | Pig | PE-Cy^TM^7 | Directly conjugated | BD biosciences | 1/200 |
| CD21 | BB6-11C9.6 | Mouse IgG1 | Pig | PE | Directly conjugated | Southern Biotech/BioNova | 1/100 |
| γδ T Lymphocytes* | MAC320 | Rat IgG2a | Pig | APC | Directly conjugated | BD biosciences | 1/100 |
| Control IgG1 Isotype | MOPC-21 | Mouse IgG1 |  | FITC | Directly conjugated | BD biosciences | 1/100 |
| Control IgG2a Isotype | G155-178 | Mouse IgG2a |  | PE-Cy^TM^7 | Directly conjugated | BD biosciences | 1/100 |
| Control IgG2b Isotype | 27-35 | Mouse IgG2b |  | Alexa Fluor® 647 | Directly conjugated | BD biosciences | 1/100 |

*Previously used in Ballester et al., 2020 (12)

**Supplementary Table 2.** Phenotypic correlation coefficients among the health-related traits.

**Supplementary Table 3.** Genetic correlation coefficients and their estimation standard errors among the health-related traits.

**Supplementary Table 4.** Description of the 32 associated SNPs with immune-cell traits, including chromosome (Chr), position in bp, associated trait, minor allele frequency, additive value, p-value and corrected p-value.

**Supplementary Table 5.** Description of the 32 associated SNPs with their predicted consequences.

**Supplementary Table 6.** List of genes differentially expressed in blood between H and L groups for T helper and T memory cell percentages considering an FDR<0.1 and FC>1.2 (i.e ǀlogFCǀ > 0.26).

**Supplementary Table 7.** Immune system processes identified in the list of differently expressed genes (FDR<0.1 and FC>1.2 (i.e ǀlogFCǀ>0.26)) in blood between H and L groups for T helper and T memory cell percentages.

**Supplementary Table 8.** List of genes differentially expressed in blood between H and L groups for percentages of γδ T cells considering an FDR<0.1 and FC>1.2 (i.e ǀlogFCǀ > 0.26).

**Supplementary Table 9.** Immune system processes identified in the list of differently expressed genes (FDR<0.1 and FC>1.2 (i.e ǀlogFCǀ>0.26)) in blood between H and L groups for percentages of γδ T cells.
